# Apolipoprotein(a) Kringle-IV Type 2 Copy Number Variation Is Associated with Venous Thromboembolism

**DOI:** 10.1371/journal.pone.0149427

**Published:** 2016-02-22

**Authors:** Elena Sticchi, Alberto Magi, Pia R. Kamstrup, Rossella Marcucci, Domenico Prisco, Ida Martinelli, Pier Mannuccio Mannucci, Rosanna Abbate, Betti Giusti

**Affiliations:** 1 Department of Experimental and Clinical Medicine, University of Florence—Atherothrombotic Disease Center, Careggi Hospital, Florence, Italy; 2 Department of Clinical Biochemistry, Copenhagen University Hospital—Herlev, Herlev, Denmark; 3 Department of Experimental and Clinical Medicine, University of Florence—SOD Patologia Medica, Center for Autoimmune Systemic Diseases, Behçet Center and Lupus Clinic, Careggi Hospital, Florence, Italy; 4 A. Bianchi Bonomi Hemophilia and Thrombosis Center—Ospedale Maggiore Policlinico, Milan, Italy; 5 Scientific Direction, Fondazione Ca’ Granda–Ospedale Maggiore Policlinico, Milan, Italy; Ottawa Hospital Research Institute, CANADA

## Abstract

In addition to the established association between high lipoprotein(a) [Lp(a)] concentrations and coronary artery disease, an association between Lp(a) and venous thromboembolism (VTE) has also been described. Lp(a) is controlled by genetic variants in *LPA* gene, coding for apolipoprotein(a), including the kringle-IV type 2 (KIV-2) size polymorphism. Aim of the study was to investigate the role of *LPA* gene KIV-2 size polymorphism and single nucleotide polymorphisms (SNPs) (rs1853021, rs1800769, rs3798220, rs10455872) in modulating VTE susceptibility. Five hundred and sixteen patients with VTE without hereditary and acquired thrombophilia and 1117 healthy control subjects, comparable for age and sex, were investigated. *LPA* KIV-2 polymorphism, rs3798220 and rs10455872 SNPs were genotyped by TaqMan technology. Concerning rs1853021 and rs1800769 SNPs, PCR-RFLP assay was used. *LPA* KIV-2 repeat number was significantly lower in patients than in controls [median (interquartile range) 11(6–17) vs 15(9–25), p<0.0001]. A significantly higher prevalence of KIV-2 repeat number ≤7 was observed in patients than in controls (33.5% vs 15.5%, p<0.0001). KIV-2 repeat number was independently associated with VTE (p = 4.36 x10^-9^), as evidenced by the general linear model analysis adjusted for transient risk factors. No significant difference in allele frequency for all SNPs investigated was observed. Haplotype analysis showed that *LPA* haplotypes rather than individual SNPs influenced disease susceptibility. Receiver operating characteristic curves analysis showed that a combined risk prediction model, including KIV-2 size polymorphism and clinical variables, had a higher performance in identifying subjects at VTE risk than a clinical-only model, also separately in men and women.

## Introduction

Lipoprotein(a) [Lp(a)] is composed of an apolipoprotein B100 molecule covalently bound to the glycoprotein apolipoprotein (a) [apo(a)]. Experimental data showed that high Lp(a) levels could contribute to promote atherosclerosis via Lp(a)-derived cholesterol entrapment in the intima, inflammatory cell recruitment, and/or the binding of pro-inflammatory-oxidized phospholipids [1–4]. Moreover, Lp(a) prothrombotic effect might also be related to the similarity of apo(a) to plasminogen, thereby interfering with plasminogen’s antithrombotic functions [5,6,7] Several studies, including a large meta-analysis, demonstrated that high circulating Lp(a) levels were consistently associated with coronary artery disease (CAD) [8–11], and represent an independent predictor of coronary artery calcification, as a marker of coronary atherosclerosis [12].

Lp(a) levels are highly heritable, thus being largely controlled by genetic variants at the *LPA* [lipoprotein, Lp(a) gene, OMIM +152200] locus [13]. In particular, the presence of a copy number variation (CNV) consisting in a variable number of kringle (K) IV type 2 repeats (KIV-2) in the *LPA* gene is the main determinant of Lp(a) levels. The National Center for Biotechnology Information reference sequence (GRCh37/hg19) contains 7 repeats of the KIV-2 domain; however, KIV-2 repeats number varies among individuals, ranging from 2 to >40 times per allele in humans [14]. The genetically determined KIV-2 repeat size contributes to 30–70% of the variation in Lp(a) levels [13,15], and affects the final size of apo(a), with larger isoforms being compromised with respect to protein folding, transport and secretion [16].

Actually, a low number of *LPA* KIV-2 repeats was associated with increased Lp(a) circulating levels (15), and influences CAD susceptibility [17].

Apart from KIV-2 repeats, single nucleotide polymorphisms (SNPs) have been described to modulate Lp(a) levels. Experimental studies showed that 93C>T (rs1853021) and 121G>A (rs1800769) SNPs in the 5’-UTR region of *LPA* gene were able to influence, decreasing and increasing gene expression respectively [18,19].

A multicenter case-control study, investigating the role of SNPs in candidate genes, identified two variants at the *LPA* locus (rs3798220, rs10455872), associated with increased Lp(a) levels, which influence CAD susceptibility [20]. Beyond the role of Lp(a) in modulating CAD risk, some studies investigated the possible association between Lp(a) levels, *LPA* genetic variants and venous thromboembolism (VTE) [21–33]. Although these studies showed conflicting results, data from a meta-analysis provided evidence for a significant association between high Lp(a) levels and VTE [34]. Moreover, high Lp(a) levels in patients with residual vein obstruction are associated with less permeable and poorly lysable plasma fibrin clots [35].

Therefore, based on the abovementioned observations and considering that Lp(a) circulating levels are under a strict genetic control, we carried out a case-control study investigating KIV-2 repeats and SNPs previously associated with Lp(a) levels (*LPA* rs1853021,rs1800769, rs3798220, rs10455872), in patients with VTE.

## Methods

### Subjects

One thousand two hundred seventy eight patients were consecutively referred to the Thrombosis Center of Milan (Italy) from January 2000 to December 2003 to be investigated for thrombophilia after a first episode of symptomatic VTE. After the exclusion of patients with antithrombin, protein C, or protein S deficiency, factor V Leiden or prothrombin G20210A polymorphism, antiphospholipid antibodies, and those in whom diagnosis of VTE was not objectively confirmed, 516 patients were included in the study (Table 1).

**Table 1:** Demographic and clinical characteristics of patients with venous thromboembolism (VTE) and control subjects.

Characteristics	VTE patients (N = 516)	Controls (N = 1117)	P
Age, years*	44 (9–80)	44 (12–84)	0.078
**Sex (male), N (%)**	199 (38.6)	477 (42.7)	0.117
**Smoking habit, N (%)**	79 (15.3)	303 (27.1)	**<0.0001**
**Diabetes, N (%)**	7 (1.4)	2 (0.2)	**0.006**
**Hypertension, N (%)**	33 (6.4)	95 (8.5)	0.165
**Dyslipidemia, N (%)**	26 (5.0)	32 (2.9)	**0.031**
**BMI, kg/m**^**2**^ *	24.6 (15.2–50.2)	23.6 (15.6–45.2)	**<0.0001**
**Hyperhomocysteinemia, N(%)**	62 (12.0)	54 (4.8)	**<0.0001**
***Transient risk factor of VTE***			
**Trauma/Prolonged Immobilization, N (%)**	54 (10.5)	**-**	**-**
**Surgery, N (%)**	52 (10.1)	**-**	**-**
**Oral contraceptives/HRT, N (%)**	112 (35.3)	111 (17.3)	**<0.0001**
***Type of VT***			
**DVT lower limb, N (%)**	236 (45.7)	**-**	**-**
**DVT upper limb, N (%)**	35 (6.8)	**-**	**-**
**Pulmonary Embolism, N (%)**	67 (13.0)	**-**	**-**
**SVT lower limb, N (%)**	106 (20.5)	**-**	**-**
**SVT upper limb, N (%)**	19 (3.7)	**-**	**-**
**Cerebral vein thrombosis, N (%)**	26 (5.0)	**-**	**-**
**Visceral vein thrombosis, N (%)**	27 (5.2)	**-**	**-**

* = median (range) values; BMI = body mass index; HRT = hormonal replacement therapy; DVT = deep vein thrombosis; SVT = superficial vein thrombosis

VTE was objectively confirmed by B-mode compression ultrasound (deep or superficial vein thrombosis) or contrast venography (deep vein thrombosis), (ventilation)/perfusion lung scan, computed tomography or angiography (pulmonary embolism), CT angiography, magnetic resonance or magnetic resonance angiography (cerebral or visceral thrombosis).

The presence of transient risk factors in the month preceding thrombosis was recorded; these were surgery, trauma, leg cast, prolonged immobilization (>10 days), oral contraceptive use or hormone replacement therapy. In the absence of the aforementioned conditions, thrombosis was considered unprovoked.

Controls were 1117 healthy individuals (477 males, 640 females), partners or friend of the whole population of patients referred to the Thrombosis Center in Milan, who volunteered to be investigated for thrombophilia and were enrolled in the same period of cases. Thrombosis and family history were excluded in controls with a structured questionnaire validated for the retrospective diagnosis of VTE [36]. Control subjects with antithrombin, protein C, or protein S deficiency, with factor V Leiden or prothrombin G20210A polymorphisms, with antiphospholipid antibodies were excluded.

All patients and controls were of Western European descent and free from overt autoimmune or neoplastic diseases. Hyperhomocysteinemia was defined as basal homocysteinemia >14.88 nmol/ml in females and >19.25 nmol/ml in males. All of them gave their written informed consent and the study was approved by the Institutional Review Board of the Fondazione IRCCS Ca’ Granda—Ospedale Maggiore Policlinico.

### DNA extraction

Genomic DNA was extracted from peripheral venous blood using FlexiGene Kit (Qiagen, Germany).

### SNPs genotyping

*LPA* +93C>T (rs1853021) and +121G>A (rs1800769) polymorphisms have been detected with PCR-RFLP analysis, as previously described [37].

*LPA* rs3798220 and rs10455872polymorphisms were genotyped by real time PCR and specific Taqman assays (Life technologies). Table 2 shows the characteristics of the 4 selected polymorphisms.

**Table 2:** Characteristics of the 4 selected *LPA* gene single nucleotide polymorphisms (SNPs).

Chr. Position	Nucleotide substitution	SNP ID	MAF dbSNP	MAF Literature	Region	Assay number or primer design	Association with Lp(a) levels
160961137	*T/C*	rs3798220	C<0.01	C = 0.02 [20]	exon 36 (cds)	C__25930271_10	Yes [20]
161010118	*A/G*	rs10455872	G = 0.09	G = 0.07 [20]	intron 25	C__30016089_10	Yes [20]
161085267	*G/A*	rs1800769	A = 0.205	A = 0.100 [44]	5’-UTR	primer design [37]	Yes [19]
							No [44]
161085295	*C/T*	rs1853021	T = 0.182	T = 0.160 [44]	5’-UTR	primer design [37]	Yes [19]
							No [45]
							Yes [44]
							Yes [46]

MAF = Minor Allele Frequency

### Kringle IV Type 2 (KIV-2) repeats evaluation

The *LPA* KIV-2 size polymorphism was genotyped by real-time polymerase chain reaction (PCR) analysis using the 7900HT Sequence Detection System (Life Technologies) according to a modified protocol of previously developed assays [17,38]. Genotyping resulted in an estimate of the total number (sum of repeats on both alleles) of KIV-2 repeats. Taqman telomerase reverse transcriptase (*TERT*) control reagent was utilized as a single-copy reference gene and also used to normalize for different concentrations of DNA in different samples. To improve precision, all samples were analyzed by the same molecular biologist, using the same calibrator and control samples. The calibrator and control samples were kindly supplied by Dr. Pia R. Kamstrup (Dpt. Clinical Biochemistry, Copenhagen University Hospital—Herlev, Denmark) [17].

### qPCR data normalization and analysis

The Ct values obtained from each plate of the "quantitative PCR" experiments were stored in.xls (excel) files and loaded into the R statistical environment by using the "read.xls" function of the "gdata" package. As a first step, for each plate, we averaged the Ct values across technical replicates for both test (*LPA* KIV-2) and control DNA (*TERT*) and we then calculated the DeltaCt (ΔCt) values between *LPA* KIV-2 and *TERT*. As a further step, in order to make CNV values comparable within and between different plates, we used the ΔΔCt approach using the ΔCt of a reference sample as control. For each plate, the ΔΔCt values and the number of KIV-2 repeats were calculated using the following formulas:

ΔΔCt=ΔCtLPAKIV-2−ΔCt Control

2^[-(ΔΔCt)]*27.

**Fig 1:**
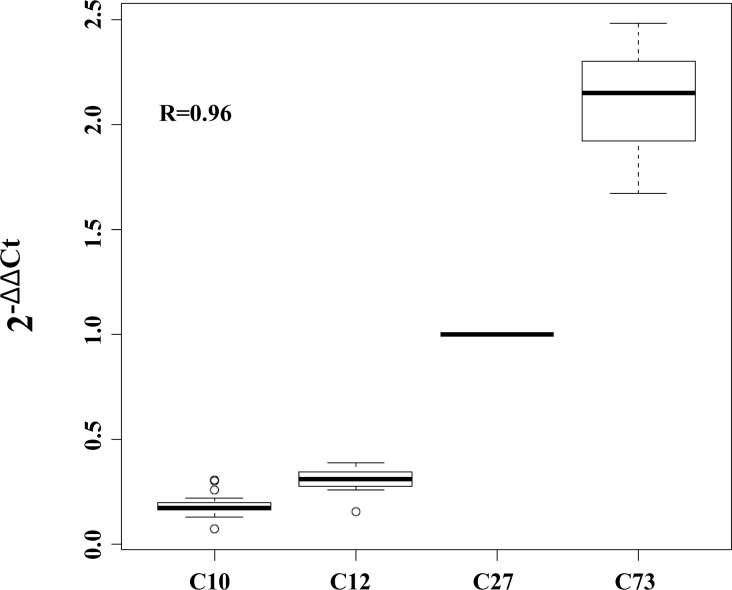
Correlation between ΔΔCt and absolute number of DNA copies of LPA exon 4. The C27 sample, previously predicted to have 27 DNA copies of this region, has been used as reference.

To this end, as a reference sample, we used the ΔCt value of the C27 sample that was previously predicted to have 27 DNA copies of the exon 4 of the *LPA* gene. In order to show the capability of the ΔΔCt approach to make data comparable within and between plates and to predict absolute number of DNA copies, we applied it to samples that were previously genotyped to have 10 (C10), 12 (C12), and 73 (C73) copies of the exon 4 of the *LPA* gene [16]. We used the raw data from 23 different plates: in each plate we genotyped the exon 4 of the LPA gene of the four characterized samples (C10, C12, C27 and C73). The results of this analysis are reported in Fig 1 and clearly show that the correlation between the ΔΔCt and the absolute number of DNA copies of the *LPA* KIV-2 is very high (R = 0.96).

All of the association analyses involving CNV were performed by using the "glm" function (general linear model) implemented in the R statistical environment using the ΔΔCt values described above as a measure of the number of DNA copies of the KIV-2 repeats in the *LPA* gene.

### Statistical analysis

Statistical analysis was performed using the SPSS package v19. Hardy–Weinberg equilibrium (HWE) was evaluated by χ2 test. Genotype distributions were compared between VTE patients and controls or among patients with different localizations of VTE by χ2 analysis. Categorical variables are expressed as frequencies and percentages. Unless otherwise indicated, continuous data are given as median and range. Comparisons of continuous variables between patients and controls were performed by the non-parametric Mann–Whitney test.

Post-hoc sample-size calculations indicated that a number of 516 patients and 1117 controls have a statistical power (β) to detect significant different average values of 97% for both ΔCt values and KIV-2 repeats, and to detect significant different percentages of genotypes of 12% for rs3798220, 12% for rs10455872, 31% for rs1853021, and 53% for rs1800769. Logistic regression analysis, adjusted for age, gender, hypertension, diabetes mellitus, dyslipidemia, smoking habit, body mass index (BMI), and oral contraceptives use was performed to estimate odds ratios (ORs) and 95% confidence intervals (CIs) for the risk of VTE associated to KIV-2 number. Haplotype analysis was performed according to methods previously reported [39]. Briefly, data files were processed in R environment, haplotype reconstruction and frequency estimation were independently performed using the PHASE v2.1 software and R package haplo.stats by Expectation-Maximization strategy (EM algorithm).

The Bonferroni correction was used for multiple testing (the five candidate polymorphisms were treated as four independent statistical tests) by multiplying the nominal P-value of each test by the number of tests conducted.

Baseline clinical characteristics were considered for the clinical-only model, whereas KIV-2 repeat number was considered for the genetic-only model. For each model, the regression parameter estimates of the independent variables were calculated and used to derive 3 different weighted equations corresponding to the clinical-only, genetic-only, and combined (genetic and clinical) models, respectively. Nonparametric receiver operating characteristic (ROC) curves were used to assess the discriminatory power of the 3 prediction algorithms to distinguish VTE cases and controls. Pairwise comparisons of the area under the curve (AUC) were performed according to DeLong et al [40]. AUC 95% confidence interval was calculated by Bootstrap approach. For each model, the best cutoff that maximized the sensitivity-specificity sum was determined and gives an indication of the optimal model’s sensitivity and specificity.

A value of p<0.05 was chosen as the cut-off level for statistical significance.

## Results

In Table 1, demographic and clinical characteristics of the 516 patients with VTE and 1117 controls are shown. Patients significantly differed from controls for smoking habit, diabetes, dyslipidemia, body mass index (BMI), and hyperhomocysteinemia. Transient risk factors and type of venous thrombosis are also reported in Table 1.

### Single nucleotide polymorphisms

Table 3 shows the genotype distributions and allele frequencies in VTE patients and controls of the four SNPs (rs3798220, rs10455872, rs1800769, and rs1853021).

**Table 3:** Genotype distribution and minor allele frequency of the 4 *LPA* single nucleotide polymorphisms (SNPs) investigated.

*LPA* SNPs	VTE patients (N = 516)	Controls (N = 1117)	p
**rs3798220**			
**TT**	505 (97.9)	1089 (97.5)	
**TC**	11 (2.1)	28 (2.5)	0.729
**CC**	-	-	
**C allele frequency**	0.011	0.013	0.775
**rs10455872**			
**AA**	472 (91.5)	1014 (90.8)	
**AG**	43 (8.3)	98 (8.8)	
**GG**	1 (0.2)	5 (0.4)	0.699
**G allele frequency**	0.044	0.048	0.612
**rs1800769 (*LPA* 121G>A)**			
**GG**	389 (75.4)	883 (79.1)	
**GA**	120 (23.3)	226 (20.2)	
**AA**	7 (1.4)	8 (0.7)	0.157
**A allele frequency**	0.130	0.108	0.083
**rs1853021 (*LPA* 93C>T)**			
**CC**	355 (68.8)	800 (71.6)	
**CT**	142 (27.5)	295 (26.4)	
**TT**	19 (3.7)	22 (2.0)	0.096
**T allele frequency**	0.174	0.152	0.110

The genotype distributions of the four polymorphisms respected the Hardy-Weinberg equilibrium in patients and controls. The minor allele frequencies of the 4 polymorphisms were similar to those expected on the basis of dbSNP and literature data in European population. No significant differences between patients and controls in genotype distribution and allele frequency for the 4 SNPs were observed by χ2 analysis (Table 3) and by both univariate and multivariate logistic regression analysis (data not shown). After haplotypes reconstruction analysis of the four SNPs in the *LPA* gene, the analysis of association with VTE by using the generalized linear model adjusted for age, gender, hypertension, smoking habit, dyslipidemia, diabetes, BMI, hyperhomocysteinemia and oral contraceptives/ use of hormone replacement therapy identified that TAAC and tagt haplotypes were significant and independent risk factors for VTE (Table 4).

**Table 4:** Haplotypes reconstruction analysis of the four SNPs in the *LPA* gene and analysis of association with VTE by using the generalized linear model (adjusted for venous thrombosis risk factors*).

Haplotypes	Frequencies in control subjects	Frequencies in VT patients	Coefficient	Standard Error	p-values
T A G C	0.691	0.655			
T A A C	0.098	0.116	0.067	0.027	**0.014**
T G G C	0.048	0.041	-0.003	0.037	0.935
T A G T	0.150	0.171	0.046	0.022	**0.033**
Rare haplotypes	0.013	0.017	0.016	0.080	0.843

SNP 1 = rs3798220; SNP 2 = rs10455872; SNP 3 = rs1800769; SNP 4 = rs1853021. SNPs in haplotypes are reported from 5’ to 3’ end of the *LPA* gene

*adjusted for: age, gender, hypertension, smoking habit, dyslipidemia, diabetes mellitus, body mass index, hyperhomocysteinemia and oral contraceptives/HRT use.

### KIV-2 repeat genetic variant

The median values and interquartile range of ΔΔCt or number of KIV-2 repeats and the prevalence of subjects with KIV-2 repeats in the different quartiles significantly differed between VTE patients and controls (Table 5).

**Table 5:** *LPA* KIV-2 repeats in VTE patients.

*LPA* KIV-2 size polymorphism	VTE patients (n = 516)	Controls (n = 1117)	P
ΔΔCt*	1.34 (0.68–2.19)	0.90 (0.13–1.55)	**<0.0001**
KIV-2 repeats number*	11 (6–17)	15 (9–25)	**<0.0001**
**KIV-2 repeat number quartiles**			
*Q1 (≤7 repeats)*, *N(%)*	173 (33.5)	173 (15.5)	**<0.0001**
*Q2 (8–12 repeats)*, *N(%)*	134 (26.0)	285 (25.5)	
*Q3 (13–22 repeats)*, *N(%)*	119 (23.1)	341 (30.5)	
*Q4 (≥23 repeats)*, *N(%)*	90 (17.4)	318 (28.5)	

*median (interquartile range)

Indeed, patients showed significantly higher ΔΔCt and significantly lower KIV-2 repeat number than controls and subjects in the lower quartile of KIV-2 repeats were significantly more prevalent in patients than in controls (Table 5). Patients with transient risk factors (n = 197) showed similar ΔΔCt and KIV-2 repeat number in comparison to that observed in patients without transient risk factors (n = 319): 1.22 (0.55–2.51) vs 1.38 (0.72–2.09) p = 0.759, and 12 (5–18) vs 10 (6–16) p = 0.738, respectively.

No differences were observed according to the type and localization of venous thrombosis: KIV-2 repeats for DVT lower limb = 11 (6–18); DVT upper limb = 11 (5–17); pulmonary embolism = 12 (5–17); SVT lower limb = 10 (6–16); SVT upper limb = 10 (8–17); cerebral vein thrombosis = 10 (5–15); visceral vein thrombosis = 10 (6–21); p = 0.991.

**Fig 2:**
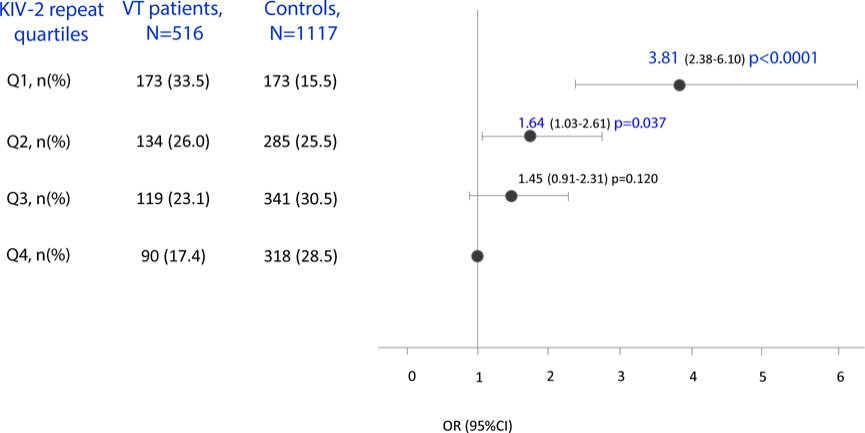
Association between KIV-2 repeat quartiles (Q1: ≤7 repeats; Q2: 8–12 repeats; Q3: 13–22 repeats; Q4: ≥23 repeats) and venous thromboembolism. Multivariate logistic regression analysis: adjusted for age, sex, hypertension, smoking habit, dyslipidemia, diabetes, BMI, hyperhomocysteinemia and oral contraceptives/HRT; Q4: reference group.

At logistic regression analysis adjusted for traditional cardiovascular risk factors, odds ratio for VTE of the subjects in the first and second quartile of KIV-2 repeats was 3.81 (95% CI 2.38–6.10, p<0.0001) and 1.64 (95% CI 1.03–2.61, p = 0.037), respectively (Fig 2). When the multivariate logistic regression analysis was separately performed in patients according to the presence of transient risk factors, odds ratio for VTE in patients with transient risk factors in the first quartile of KIV-2 repeats was 3.13 (95% CI 1.95–5.03), p<0.0001; odds ratio for VTE in patients without transient risk factors in the first and second quartile of KIV-2 repeats was 3.75 (95% CI 2.46–5.71), p<0.0001, and 2.25 (95% CI 1.50–3.38), p<0.0001.

At the general linear model analysis adjusted for age, sex, hypertension, smoking habit, dyslipidemia, diabetes, BMI, hyperhomocysteinemia and oral contraceptives/HRT use with VTE as dependent variable and number of KIV-2 repeats as independent variable, KIV-2 repeat number was a significant and independent determinant of the disease (coefficient = -0.004, standard error = 0.0007, p = 4.36 x 10^−9^), also after Bonferroni correction (p = 2.18 x 10^−8^).

This finding has been separately confirmed in men and women (data not shown).

**Fig 3:**
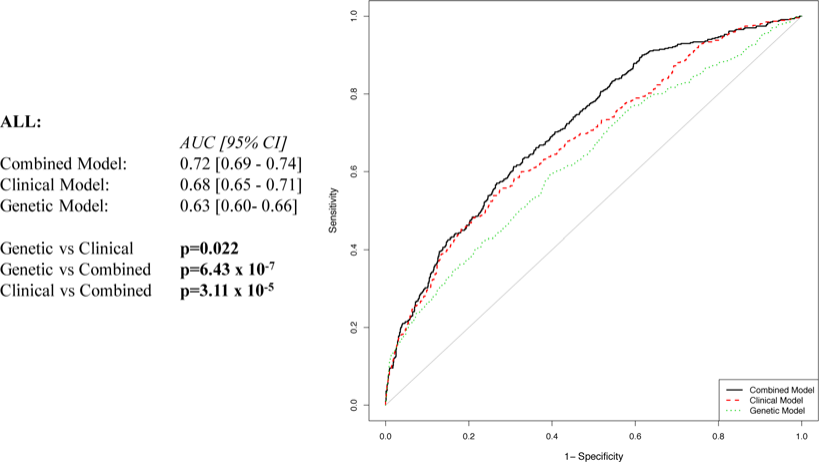
Receiver operating characteristic curve for association with venous thromboembolism in the whole population. Variables included in the models: Genetic = KIV-2 repeats; Clinical = age, sex, hypertension, smoking habit, dyslipidemia, diabetes, BMI, hyperhomocysteinemia, and oral contraceptives/HRT; Combined = Genetic + Clinical variables.

In addition to the combined (clinical and genetic) model, a model including only clinical characteristics (clinical-only model) and a model including KIV-2 repeat number (genetic-only model) were built using multivariable logistic regression analyses. The regression parameter estimates of the independent variables were used to derive 3 different weighted equations. We then compared the predictive performance of these models by ROC curve analyses (Fig 3).

**Fig 4:**
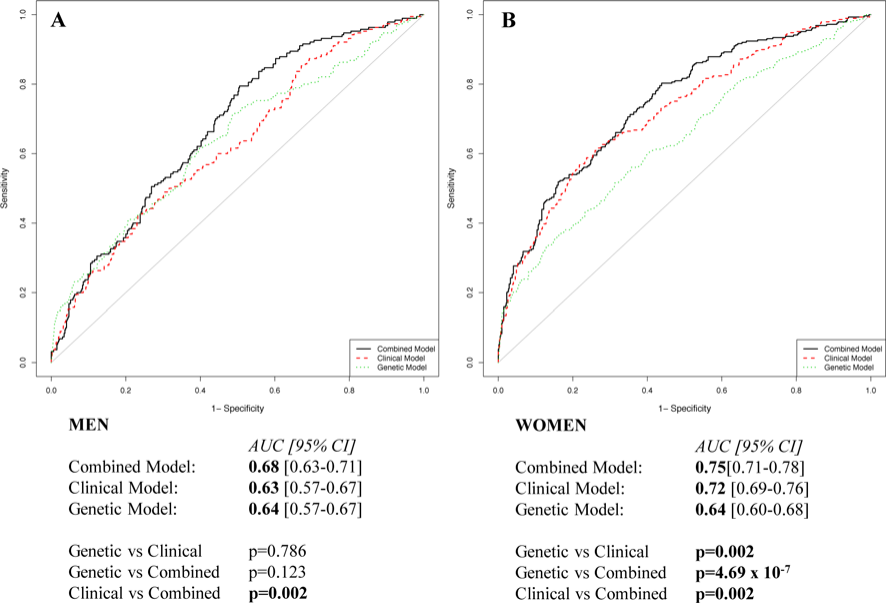
**Receiver operating characteristic curve for association with venous thromboembolism in men (A) and women (B).** Variables included in the models: Genetic = KIV-2 repeats; Clinical = age, hypertension, smoking habit, dyslipidemia, diabetes, BMI and hyperhomocysteinemia for men, and age, hypertension, smoking habit, dyslipidemia, diabetes, BMI, hyperhomocysteinemia, and oral contraceptives/HRT for women; Combined = Genetic + Clinical variables.

A higher predictive performance of the clinical-only with respect to the genetic-only model [AUC, 0.68 (95%CI, 0.65–0.71) vs 0.63 (95%CI, 0.60–0.66), p = 0.022] was observed. Nevertheless, we evidenced a greater predictive power of the combined model [AUC, 0.72 (95%CI, 0.69–0.74)] in comparison to genetic-only and clinical-only model (p = 6.43 x10^-7^ and p = 3.11 x10^-5^, respectively). The same analysis was also separately performed in men and women (Fig 4), finding that the clinical-only and genetic-only models were able to similarly discriminate between patients and control men [AUC, 0.63 (95% CI, 0.57–0.67) vs 0.64 (95% CI, 0.57–0.67), respectively; p = 0.786]. The combined model [AUC, 0.68 (95% CI, 0.63–0.71)] had significantly greater power to discriminate patients than the clinical-only model in men (p = 0.002) (Fig 4). Concerning women, the predictive performance of the genetic-only model was similar to that observed in men [AUC, 0.64 (95% CI, 0.60–0.68)]. The clinical-only [AUC, 0.72 (95% CI, 0.69–0.76)] significantly differed from the genetic-only model in women (p = 0.002). Moreover, the combined model [AUC, 0.75 (95% CI, 0.71–0.78)] had significantly greater power to discriminate patients than both clinical-only and genetic-only models (p = 0.002 and p = 4.69 x 10^−7^, respectively) (Fig 4).

## Discussion

In the present study we demonstrated, for the first time, that low *LPA* KIV-2 repeat number, known to explain 30–70% of Lp(a) phenotypic variance [13], significantly and independently predispose to VTE.

Moreover, two *LPA* haplotypes (TAAC and TAGT) rather than the individual SNPs (rs3798220, rs10455872, rs1800769, and rs1853021) mildly influenced disease predisposition.

Several data are available on the contribution of the genetic variants studied in this paper to coronary artery disease. In particular, a low number of KIV-2 repeats has been associated with an increased risk of myocardial infarction [14,17] and angiografically documented coronary artery stenosis >50% [14,28]. The rs3798220 and rs10455872 SNPs have been associated with CAD and cumulatively explained 36% of variation in Lp(a) levels [20]. Both KIV-2 repeats and rs10455872 variants substantially improved myocardial infarction and coronary heart disease risk prediction [14].

On the contrary, scarce information on the association of the genetic variants evaluated in this study with VTE is available.

High Lp(a) levels are associated with increased risk of VTE [23,24,28,30]. A meta-analysis of 6 case-control studies showed a statistically significant association between high Lp(a) levels and the occurrence of VTE [34].

Our data on the role *per se* of the rs3798220, rs10455872 polymorphisms are consistent with those observed in previous studies demonstrating no influence on VTE predisposition [29,30].

However, the observation that two haplotypes are associated with VTE warrants further studies on a high number of selected tagSNPs able to allow the evaluation of a higher number of alleles than those enabled by the present SNPs selected on the basis of previous literature data showing their association with Lp(a) levels.

Our major finding is that low *LPA* KIV-2 repeat number is significantly and independently associated with VTE. This is in contrast with those data stemming from two white Danish general populations [28]. These conflicting data might be due to population characteristics: our patients were affected by VTE without hereditary and acquired thrombophilia and were on average younger than the participants in the Danish studies. Moreover, the KIV-2 repeat number is extremely variable among different populations ([20,28,41], present study), and might, at least in part, influence the different results obtained in this study with respect to the Danish population [28].

In fact, according to the KIV-2 repeat number the different populations also showed very broad distribution of Lp(a) circulating levels [42]. Therefore, the impact of KIV-2 size polymorphism on clinical phenotype might differ across populations possibly due to the different prevalence of low number of repeats, and to their interaction with other genetic and environmental factors [13,34].

On the other hand, post-hoc combined analyses of extreme KIV-2 repeat numbers in the same Danish populations demonstrated a VTE increased risk for repeats <6^th^ percentile [OR = 1.3 (95%CI 1.0–1.7)].

Present data demonstrating that the combined (clinical and genetic) model had significantly greater power to discriminate VTE than the clinical only and genetic only models, by both ROC curve, suggest the opportunity to add the genetic information among determinants traditionally used to frame the individual VTE risk profile.

Our patients were affected by different types of venous thrombosis (superficial, deep, visceral and cerebral venous thrombosis). In literature, it has been reported that different types of venous thrombosis might exhibit, at least in part, distinct underlying mechanisms [43]. In the present study, no differences were observed in KIV-2 repeat according to the different types and localizations of venous thrombosis, thus suggesting the contribution of the polymorphism in influencing a common pathogenetic mechanism.

Limitations of our study are represented by the lack of Lp(a) circulating levels in patients and controls, and of an independent replication cohort. Concerning findings on *LPA* SNPs, our study did not reach the adequate statistical power to exclude their role in influencing VTE.

In conclusion, our results support the relevance of KIV-2 size polymorphism in predicting venous thromboembolism. Our data prompt the need of further studies investigating the clinical utility of this genetic variant in the management of VTE patients.
